# Cyclooxygenase-1 and Prostacyclin Production by Endothelial Cells in the Presence of Mild Oxidative Stress

**DOI:** 10.1371/journal.pone.0056683

**Published:** 2013-02-18

**Authors:** Alice Toniolo, Carola Buccellati, Christian Pinna, Rosa Maria Gaion, Angelo Sala, Chiara Bolego

**Affiliations:** 1 Department of Pharmaceutical and Pharmacological Sciences, University of Padova, Padova, Italy; 2 Dipartimento di Scienze Farmacologiche e Biomolecolari, Università di Milano, Milano, Italy; 3 IBIM, Consiglio Nazionale delle Ricerche, Palermo, Italy; Instituto de Biofisica Carlos Chagas Filho, Universidade Federal do Rio de Janeiro, Brazil

## Abstract

This study aimed at evaluating the relative contribution of endothelial cyclooxygenase-1 and -2 (COX-1 and COX-2) to prostacyclin (PGI_2_) production in the presence of mild oxidative stress resulting from autooxidation of polyphenols such as (-)-epigallocatechin 3-gallate (EGCG), using both endothelial cells in culture and isolated blood vessels. EGCG treatment resulted in an increase in hydrogen peroxide formation in human umbilical vein endothelial cells. In the presence of exogenous arachidonic acid and EGCG, PGI_2_ production was preferentially inhibited by a selective COX-1 inhibitor. This effect of selective inhibition was also substantially reversed by catalase. In addition, EGCG caused vasorelaxation of rat aortic ring only partially abolished by a nitric oxide synthase inhibitor. Concomitant treatment with a selective COX-1 inhibitor completely prevented the vasorelaxation as well as the increase in PGI_2_ accumulation in the perfusate observed in EGCG-treated aortic rings, while a selective COX-2 inhibitor was completely uneffective. Our data strongly support the notions that H_2_O_2_ generation affects endothelial PGI_2_ production, making COX-1, and not COX-2, the main source of endothelial PGI_2_ under altered oxidative tone conditions. These results might be relevant to the reappraisal of the impact of COX inhibitors on vascular PGI_2_ production in patients undergoing significant oxidative stress.

## Introduction

Arachidonic acid is metabolized by cyclooxygenase (COX) isoforms to form a number of tissue-specific mediators such as prostacyclin (PGI_2_) and thromboxane A_2_ (TXA_2_), which represent functionally antagonistic vasoactive prostanoids regulating several aspects of vascular biology. There are two main COX isoforms: COX-1 is constitutively expressed in most tissues and mediates basal physiological functions, while COX-2 is induced by various stimuli, such as inflammatory cytokines, thus being mostly associated with pathological conditions [1].

Although endothelial cells constitutively express COX-1 [2], there is now a general agreement that PGI_2_ in vascular endothelium is generated mainly by COX-2, probably as a result of COX-2 expression induced by laminar flow shear stress [3]. This finding has assumed a great relevance after the reports of increased thromboembolic death associated with the use of COXIBs [4], leading to the hypothesis that the cardiovascular risk associated with the use of COXIBs is the result of their selective inhibition of the synthesis of cardio-protective PGI_2_, leaving unopposed the platelet-derived, COX-1 dependent, pro-thrombotic lipid mediator TXA_2_ [5].

It is widely known that COX enzymes are bi-functional proteins endowed with both cyclooxygenase (COX) and peroxidase (POX) activities, and that hydroperoxides are required for the first heme oxidation at the POX site of the enzyme [6]. Consistently, biochemical studies of COX activity using purified enzymes clearly demonstrated that COX-1 requires a higher peroxide tone than COX-2 to be activated [7], suggesting that hydroperoxide availability could enhance prostanoid production via COX-1 rather than COX-2. We previously observed that COX isoform activity in HUVECs resembles that of isolated enzymes, as we provided evidence that PGI_2_ production by endothelial cells undergoing different modalities of oxidative stress is mediated by COX-1, abundantly expressed by these cells, and not by COX-2 [8]. Although a large body of data suggested that PGI_2_ production by endothelium could be positively [9] or negatively [10], [11] affected by free radicals, this evidence was lacking informations on the relative contribution of COX isoforms to PGI_2_ production.

Epigallocatechin-3-gallate (EGCG), the main catechin of green tea, is known to undergo auto-oxidation and generate reactive oxygen species (ROS) [12], which in turn has been reported it may cause cytoprotective effects [13] and endothelium-dependent relaxation [14]. There is no general agreement on the role of ROS produced by polyphenols in cell culture medium as recently reviewed [15], however, unpublished data from our laboratory clearly indicated an increase in ROS production by human neutrophils treated with a number of grape-derived polyphenols in phosphate buffer saline solution; additional evidence is available that EGCG elicits contraction of isolated aorta in hypertensive rats and this effect is mediated by ROS production, which in turn leads to vasoconstrictive prostanoid release [16].

Based on the consideration that ECGC may affect vascular tone through ROS production, and since endothelial COX activity is modulated by hydroperoxide tone, we used the ability of EGCG to generate low concentrations of hydrogen peroxide as a pharmacological tool for evaluating the effects of ROS on endothelial PGI_2_ production in the presence of selective COX-1 or COX-2 inhibitors, using both isolated HUVECs and *ex vivo* models of endothelial function. We found that ROS produced by EGCG lead to the production of endothelial PGI_2_ by derived by the constitutive COX-1 isoform.

## Materials and Methods

The investigation conforms to the *Guide for the Care and Use of Laboratory Animals* published by the U.S. National Institutes of Health (Bethesda, MD, USA; NIH Publication No. 85-23, revised 1996) and the study was approved by the local Ethic Committee at the Dipartimento di Scienze Farmacologiche e Biomolecolari.

### Animals

Male Sprague-Dawley rats (2 mo old; Charles River, Calco, Italy), initial weight 200–225 g, were used. The animals were housed in a conditioned environment (22±1°C, 55±5% relative humidity, 12-h light/12-h dark cycle), with free access to standard laboratory chow and tap water.

### Vasorelaxation studies

After anesthesia (Pentotal Sodium, 60 mg.kg−1, i.p., Zootecnica di S.Donato, MI, Italia), rats were sacrificed by exsanguination. Thoracic aorta was carefully removed, cleaned of fat and connective tissue, and cut into 5- to 6-mm rings. Vessels were suspended in 5-ml organ baths containing Krebs-Henselheit solution (KHS) at 37°C, continuously bubbled with 95% O_2_ and 5% CO_2_. KHS had the following composition (mM): 118 NaCl, 4.7 KCl, 1.2 KH_2_PO_4_, 1.1 MgSO_4_, 2.5 CaCl_2_, 25 NaHCO_3_, and 5.5 glucose; pH 7.4. The rings were connected to isometric tension transducers (Fort 10; World Precision Instruments, Sarasota, FL, USA) coupled with a digital recording system (PowerLab 8SP; ADInstruments, Colorado Springs, CO, USA). Vascular tissues were equilibrated for 30 min and contracted with 10^−5^ M noradrenalin (NA) to develop a maximal response. Preparations were then washed with fresh KHS, and the equilibration period was allowed to continue for a further 30 min. Experiments were carried out on tissues precontracted with NA to 60% of maximal contraction (EC_60_ = 10^−7^ M). The endothelium was considered functional because relaxation of precontracted vessels to 10^−5^ M acetylcholine was ≥80%. After equilibration, relaxation by increasing concentrations (0.1–100 µM) of EGCG was performed to obtain cumulative concentration-response curves. Selected experiments were performed in rings treated with 100 µM L-NAME and/or with the selective COX-1 inhibitor SC560 (10 nM) and COX-2 inhibitor SC236 (10 nM) before NA contraction. All reagents were purchased from Sigma (St Louis, MO, USA) if not otherwise specified. SC560 was from Cayman Chemical (Ann Arbor, Michigan, USA) and SC236 was kindly provided from Searle (St. Louis, MO, USA).

### Cell culture

Human umbilical vein endothelial cells (HUVECs) obtained as previously published [17], were grown in medium 199 (M199, Invitrogen, S. Giuliano Milanese, Milan, Italy) supplemented with 15% FCS (Euroclone; Pero, Milan, Italy), gentamicin (40 g/ml, Invitrogen), endothelial cell growth factor (25 g/ml), and heparin (100 g/ml), at 37°C in a humidified 5% CO_2_ atmosphere. Cells were identified as endothelial by their morphology and the presence of CD31-related antigen. All experiments were performed on cells at the second passage. HUVECs were seeded at equal density in 6-well plates (3×10^5^/well). For experiments, cells were incubated in M199 supplemented with 5% FCS. Selected experiments were carried out in the presence of EGCG (1–100 µM), superoxide dismutase (SOD; 0.2 U/ml), catalase (CAT; 300 U/ml), diphenyleneiodonium chloride (DPI, 1 µM), arachidonic acid (10 µM; Cayman Chemical), SC560 (1–100 nM), or SC236 (1–100 nM). Inhibitors were added 15 min before the stimulus.

### ROS concentration

ROS levels in cells treated with EGCG (10–100 µM) were determined by flow-cytometry (Beckman Coulter Epics XL Flow Cytometer) using 2′,7′-dichlorfluorescein-diacetate (DCFH-DA), a cell membrane-permeable fluorogenic probe. The acetate groups of this probe are enzymatically cleaved inside living cells. The probe can then be oxidized by intracellular oxidants (ROS) to give a product, DCF, which emits a strong, green fluorescence (λ_ex_ = 504 nm; λ_em_ = 529 nm). The fluorescence intensity increases in proportion to the level of cellular oxidants and is expressed as percent increase *vs* basal values. ECGC ability to spontaneously release ROS was also tested by incubations in M199 supplemented with 5% FCS in the presence of the ROS-sensitive probe; hydrogen peroxide (100 µM), was used as a ROS to induce the oxidation of the probe. The possibility that AA itself could spontaneously generate ROS was also tested by incubating endothelial cells (5–60 min) with AA at different concentrations (0.1–10 µM), in the absence or presence of EGCG.

### Determination of PGI_2_ production

After incubation, the culture medium or the perfusate of EGCG-treated aortic were collected and centrifuged at 10,000 *g* for 5 min. 6-keto PGF_1α_, the stable hydrolysis product of PGI_2_, was measured with specific EIA kits (Cayman Chemical) according to the manufacturer's instructions.

### Statistical analysis

Data were obtained from 3 to 6 independent experiments, each one carried out in duplicate or triplicate determinations. Comparison between groups was performed by ANOVA followed by Sheffè's test for multiple comparisons. Values of *P*≤0.05 were considered statistically significant.

## Results

### ECGC increases hydrogen peroxide production in HUVECs

**Figure 1:**
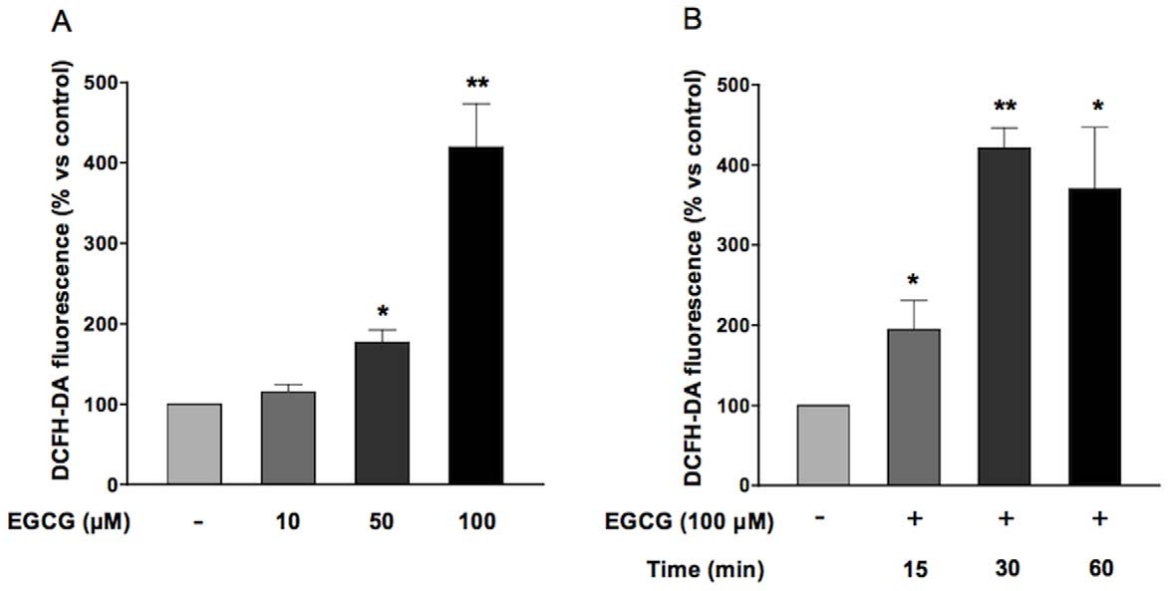
Effect of EGCG on intracellular ROS production. A) HUVECs were treated with EGCG (10, 50, 100 µM) for 30 minutes. B) HUVECs were treated with EGCG 100 µM for increasing times (15, 30, 60 min). Values are expressed as percentage ± SE (n = 14, A; n = 5, B) of the amount of ROS generated under basal conditions. *P<0.05, **P<0.1 *vs* control.

**Figure 2:**
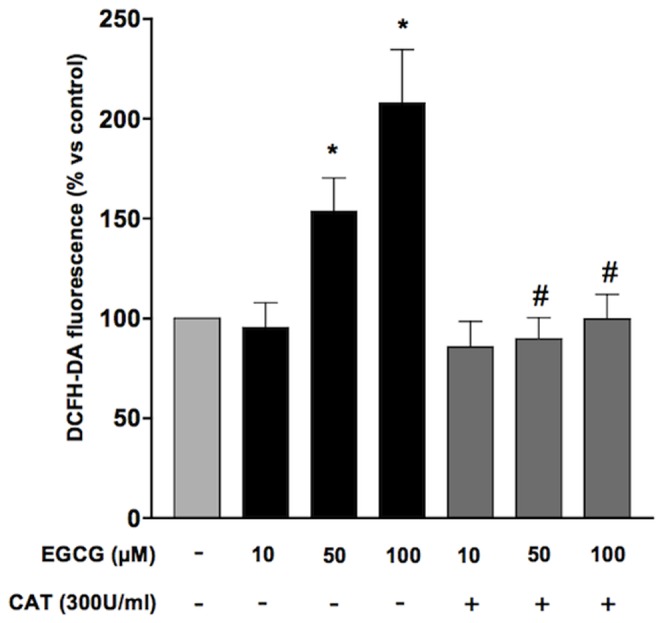
Effect of catalase on EGCG-induced ROS production. HUVECs were pre-incubated with catalase (300 U/ml) and treated with EGCG 100 µM for 30 min. Values are expressed as percentage ± SE (n = 3) of the amount of ROS generated under basal conditions. *P<0.05 *vs* control and ^#^ *P*<0.05 *vs* cells treated with EGCG.

**Figure 3:**
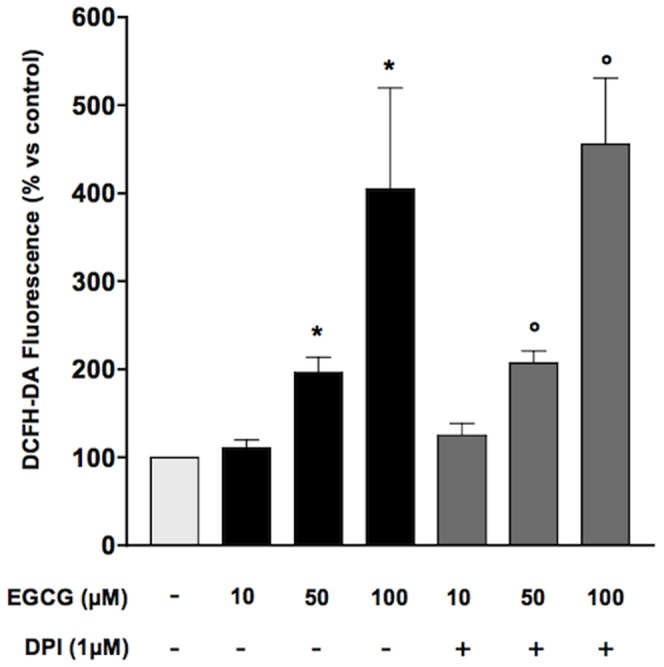
Effect of DPI on EGCG-induced ROS production. HUVECs were pre-incubated with DPI 1 µM and treated with EGCG 100 µM for 30 min. Values are expressed as percentage ± SE (n = 3) of the amount of ROS generated under basal conditions. *P<0.05 *vs* control and ^#^ *P*<0.05 *vs* cells treated with EGCG.

In order to evaluate the effect of oxidative stress, HUVECs grown in medium 199 added with 5% FCS were treated with increasing concentrations (10–100 µM) of epigallocatechin-3-gallate for different times (15–60 min) in the presence of DCFH-DA. EGCG generated an increase in ROS formation, peaking at 100 µm and 30 minutes (Figs. 1A and B). Pre-treatment of HUVECs with 300 U/ml catalase, the enzyme responsible for hydrogen peroxide breakdown, abolished the increase in ROS production triggered by ECGC (Fig. 2). Conversely, treatment of HUVECs with either the NADPH oxidase inhibitor diphenyleneiodonium (DPI; 1 µM)(Fig. 3) or superoxide dismutase (SOD; 0.2 U/ml)(data not shown) did not affect ROS production in the presence of ECGC, ruling out the involvement of endogenous enzymatic systems such as NADPH oxidase. Taken together, these data suggest that short-term treatment with EGCG increases oxidant tone in endothelial cells, mostly inducing H_2_O_2_ production by the molecule itself, independent from NADPH oxidase. Indeed, when incubated with cells in DPBS, EGCG did not result in ROS formation in HUVECs, while the simple incubation of DCFH-DA in cell culture media, in the absence of cells, resulted in conversion of the probe into the fluorescent, ROS-generated form (data not shown), confirming that medium and/or FCS components are required for EGCG autooxidation [18].

### Effect of selective COX-1 and COX-2 inhibitors on the production of PGI_2_ in the presence of ECGC in HUVECs

**Figure 4:**
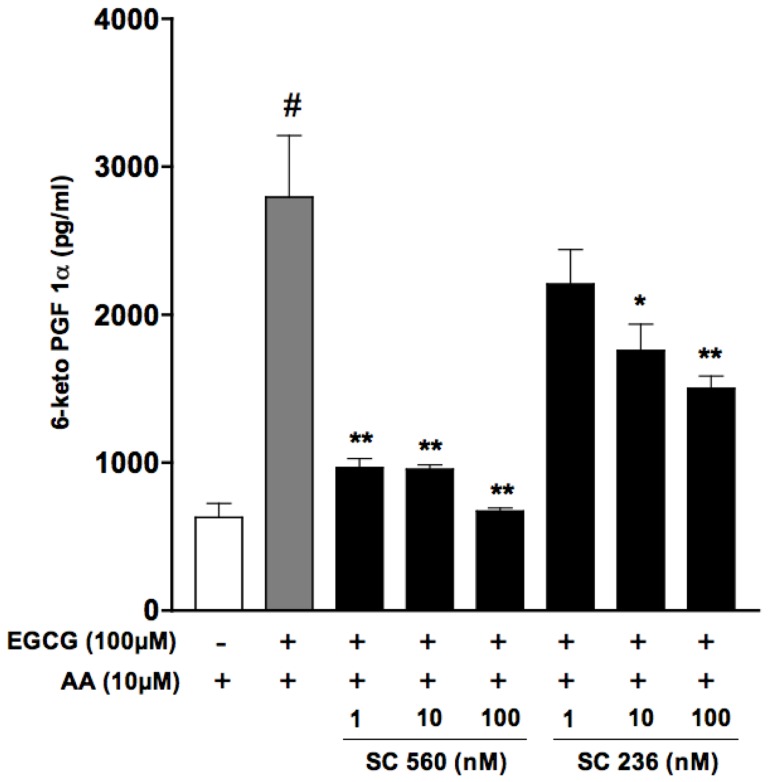
Effect of treatment with selective COX-1 (SC560) or COX-2 (SC236) inhibitors toward PGI_2_ production in HUVECs incubated with EGCG. Cells were incubated in medium 5% FCS in the presence of increasing concentrations of SC560 or SC236 (1–100 nM); 10 min after treatment with inhibitors, cells were added with arachidonic acid (10 µM) and EGCG (100 µM) for 30 min. PGI_2_ was evaluated as 6-keto-PGF_1α_ in aliquots of cell supernatants using a specific EIA. Values are expressed as pg/ml ± SE (*n* = 3) of 6-keto-PGF_1α_. # P<0.01 vs AA alone; *P<0.05, **P<0.01 *vs.* (AA+EGCG).

**Figure 5:**
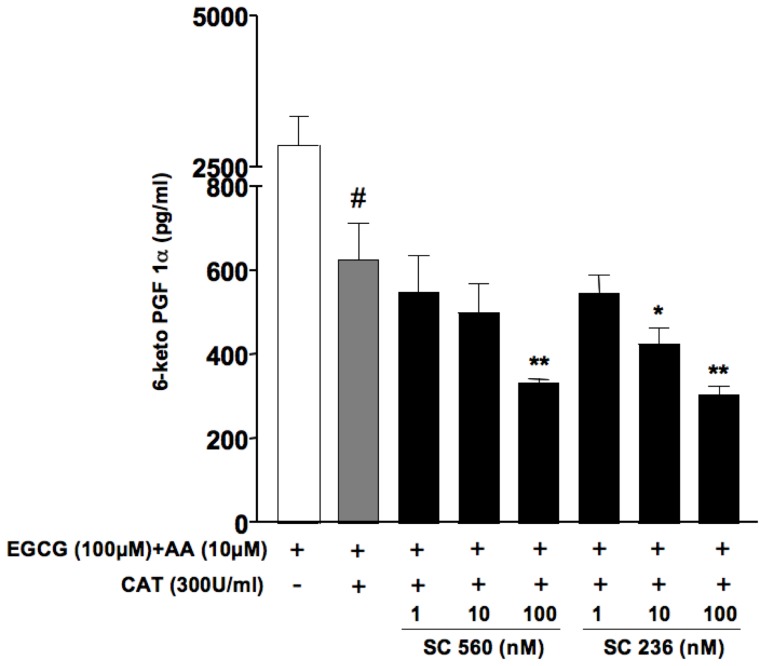
Effect of treatment with selective COX-1 (SC560) or COX-2 (SC236) inhibitors toward PGI_2_ production in HUVECs incubated with EGCG, in the presence of catalase. Cells were incubated in medium 5% FCS in the presence of increasing concentrations of SC560 or SC236 (1–100 nM); 10 min after treatment with inhibitors, cells were treated with catalase (300 U/ml) for 15 min. Afterward, arachidonic acid (10 µM) and EGCG (100 µM) were added for 30 min. PGI_2_ was evaluated as 6-keto-PGF_1α_ in aliquots of cell supernatants using a specific EIA. Values are expressed as pg/ml ± SE (*n* = 3) of the 6-keto-PGF_1α_. #P<0.01 vs (EGCG+AA); *P<0.05 and **P<0.01 *vs.* (EGCG+AA+catalase).

A lower availability of substrates or hydroperoxides may result in preferential activation of COX-2, whereas an higher “hydroperoxide tone” as well as higher arachidonic acid concentrations may direct the production of COX metabolites through the activity of COX-1. Because of the effects of EGCG on ROS formation, resulting in an increased “hydroperoxide tone” within endothelial cells, we compared the effect of the selective COX-2 inhibitor SC236 (1–100 nM) and the selective COX-1 inihibitor SC560 (1–100 nM) on PGI_2_ production in HUVECs treated with ECGC in the presence of exogenous arachidonic acid. Exogenous arachidonic acid (10 µM) per se caused a modest but significant increase in ROS formation in endothelial cells. Nevertheless this increase was negligible when compared to ROS formation associated to the incubation with EGCG (data not shown). PGI_2_ formation by endothelial cells in the presence of AA was indeed significantly enhanced upon incubation with EGCG, an increase that was potently and completely inhibited by the COX-1 inhibitor SC560, while the COX-2 inhibitor SC236 caused a rather limited (about 50%) inhibition of PGI_2_ production (Fig. 4), at a concentration (100 nM) 20-fold higher than its reported IC_50_ for COX-2. In agreement with the hypothesized role of ECGC-derived hydrogen peroxide in PGI_2_ biosynthesis, PGI_2_ production in HUVECs treated with ECGC in the presence of catalase was significantly lower with respect to that observed in the absence of catalase (0.626±0.087 ng/ml ECGC+AA+catalase *vs* 2.79±0.42 ng/ml ECGC+AA) and was concentration-dependently inhibited by both SC236 and SC560 within the same range of concentrations (Fig. 5), suggesting that, as previously published [8], in endothelial cells the basal production of PGI_2_ involves both COX-1 and COX-2 activities.

### Effect of selective COX-1 and COX-2 inhibitors on ECGC-induced vasodilation in isolated aortic rings

To further explore the possible consequences of ECGC-produced ROS on the activity of the two cyclooxygenase isoforms in endothelial cells, we used an *ex vivo* model of endothelial function.

**Figure 6:**
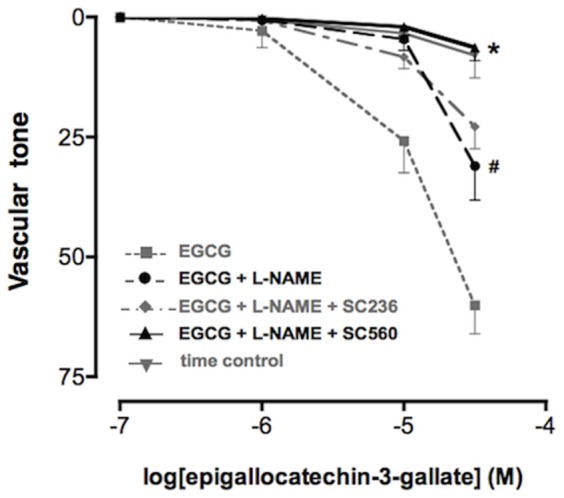
Vasorelaxing response to EGCG in the presence of selective COX-1 and COX-2 inhibitors in intact rat aortic rings. Increasing concentrations of EGCG were added to tissues pre-contracted with 10 µM NA and preincubated with L-NAME 100 µM alone or in the presence of either the selective COX-2 inhibitor SC236 10 nM or the selective COX-1 inhibitor SC560 10 nM. # *P*<0.01 (EGCG+L-NAME) *vs* (EGCG); * *P*<0.01 (EGCG+L-NAME+SC560) *vs* (EGCG+L-NAME).

**Figure 7:**
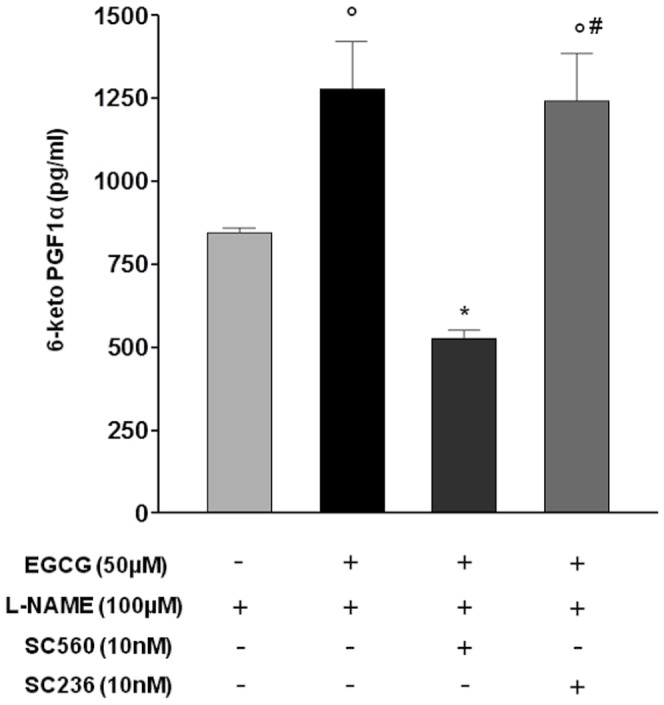
PGI_2_ production in perfusate from aortic rings treated with EGCG in the presence of selective COX-1 and COX-2 inhibitors. Samples from EGCG-treated aortic rings preincubated with L-NAME alone or in the presence of either the selective COX-2 inhibitor SC236 or the selective COX-1 inhibitor SC560 were collected and PGI_2_ was evaluated as 6-keto-PGF_1α_. Values are expressed as pg/ml of perfusate. °*P*<0.01 *vs* L-NAME; *P<0.01 *vs* (EGCG+L-NAME); #*P*<0.01 *vs* (EGCG+L-NAME+SC560).

As previously reported [19] ECGC induced a concentration-dependent (0.1–50 µM) vasorelaxation in NA-precontracted aortic rings that was only partially inhibited by preincubation with L-NAME (100 µM)(Fig. 6). Concentration-response curves with EGCG were carried out up to the concentration of 50 µM, already resulting in a consistent vasorelaxation of >50% in all the preparations tested. Furthermore, no arachidonic acid was added to the isolated organ preparations, as arachidonic acid was likely to be made available by the multicellular environment represented by the isolated organ undergoing smooth muscle contraction. Indeed mechanotransduction is known to activate the phospholipase A_2_ involved in arachidonic acid release [20], [21]. In order to determine if arachidonic acid metabolites were involved in the ECGC-induced, NO-independent vasorelaxation, tissues were pretreated with either SC236 or SC560 in the presence of L-NAME, resulting in the complete inhibition of the response to ECGC only in the presence of the COX-1 inhibitor SC560 (10 nM) but not of the COX-2 inhibitor SC236 (10 nM), suggesting a critical involvement of COX-1-derived metabolites in EGCG-mediated vasorelaxation. In strict agreement with functional data, EGCG resulted in a significant increase in PGI_2_ concentrations into the rat aorta perfusate obtained at the end of the concentration-response experiments, and such increase in the concentrations of the vasodilating PGI_2_ was completely prevented by the pretreatment with SC560 (10 nM) but not by SC236 (Fig. 7).

## Discussion

In the present study we provide evidence that, in the presence of enhanced hydroperoxide tone due to hydrogen peroxide generation by EGCG, only the selective COX-1 inhibitor SC560, but not the selective COX-2 inhibitor SC236 impaired endothelial PGI_2_ production in intact vessels, suggesting that ROS production affects endothelium-mediated vasodilation through the production of COX-1-derived PGI_2_.

PGI_2_, the main product of COX in the endothelium, exerts a variety of beneficial effects on endothelial integrity and functionality, acting *per se* as well as counteracting the pro-thrombotic and pro-adhesive properties of platelet-derived thromboxane. The withdrawal from the market of the selective COX-2 inhibitor rofecoxib after evidence of an increased cardiovascular risk associated with the use of the drug, fostered research aimed at understanding the mechanisms underlying the cardiovascular toxicity of anti-inflammatory drugs. The main accepted hypothesis, recently revised in an editorial on Science [22], is that the selective inhibition of endothelial COX-2-derived PGI_2_, leaving platelet COX-1-derived thromboxane unaffected, was responsible for the enhanced risk of thromboembolic events in patients treated with COXIBs. However, this so called “*imbalance theory*” minimizes the role of endothelial COX-1, the housekeeping isoform of cyclooxygenase which is abundantly expressed in endothelial cells.

Because COX-1 and COX-2 activity are differentially regulated by several factors, such as the availability of substrate (i.e. arachidonic acid, AA) [23], [24], [25], [26] and/or hydroperoxides [7], we hypothesized that conditions may occur that alter the relative contribution of each COX isoform to the overall PGI_2_ production, and we previously reported that, in the presence of exogenous arachidonic acid and hydroperoxides (12-HpETE) or in co-cultures with activated platelets providing an endogenous source of 12-HpETE [8], PGI_2_ production by endothelial cells appears to be mediated mainly by COX-1 and not by COX-2. Based on these observations, with the present study we further explored the possible role of oxidative stress on PGI_2_ production using EGCG autooxidation as a mild source of intracellular oxidative stress, mimiking long lasting oxidative stress in inflamed tissues, both in cultured human endothelial cells and in an *ex-vivo* model of endothelial function. Indeed EGCG might undergo auto-oxidation and may be a source of reactive oxygen species (ROS) [12], and ROS generation by EGCG, at concentrations similar to those used in our study, have been reported to contribute to cytoprotective effects [13] as well as endothelium-dependent relaxation [14]. More recently, Auger et al. [27] demonstrated that ROS from EGCG induce endothelium-dependent NO-mediated relaxation of coronary artery rings through Akt-dependent activation of eNOS, but the real contribution of ROS to the long-term *in vitro* effects of polyphenols [18] is still under debate.

It must be stressed that epigallocatechin gallate (EGCG) in our study was used at high concentration only to take advantage of its low level of spontaneous intracellular generation of reactive oxygen species together with its extremely low cellular toxicity, and our data confirmed that ECGC resulted in ROS formation within human endothelial cells, but it is difficult that there may be therapeutic implications about this kind of activity of EGCG, given the high concentrations required. In the present study, the only reactive oxygen species formed after treatment of human endothelial cells with EGCG was hydrogen peroxide, since catalase, but not superoxide dismutase, was able to abrogate EGCG-generated ROS. While a simple incubation of EGCG in tissue colture media generated a low and long lasting production of ROS, the NADPH inhibitor DPI did not decrease ROS production in EGCG-treated cells, confirming that oxidative enzyme activation was not involved in ROS formation by EGCG.

In line with studies using purified COX enzymes that indicate a differential sensitivity of COX isoforms to hydroperoxide activation, with COX-1 activation requiring higher peroxide concentration than COX-2, we demonstrated that in the presence of EGCG COX-1 is the main isoform responsible for PGI_2_ production by the endothelium. Indeed, in HUVEC challenged with EGCG the selective COX-1 inhibitor SC560 resulted more potent and effective than the selective COX-2 inhibitor SC236 in inhibiting PGI_2_ production. On the contrary, in the presence of catalase suppressing the mild oxidation induced by EGCG, both SC236 and SC560 negatively affected the residual production of PGI_2_ with similar potency and efficacy, suggesting that COX-1 becomes the main isoform responsible for endothelial PGI_2_ production only under conditions of increased hydroperoxide availability such as with EGCG or, pathophysiologically, in the presence of increased oxidative stress such as in diabetes or in the presence of activated platelets. In further agreement with these results, in a well-established *ex vivo* model of endothelial function, we demonstrated that EGCG up to 50 µM induced a concentration-dependent vasodilation, which was partially mediated by PGI_2_ and blocked by the selective COX-1 inhibitor SC560 but not by the COX-2 selective SC236. The determination of the hydrolysis product of PGI_2_ in the isolated organ superperfusion buffer at the end of the concentration-response curves with EGCG also showed increased concentrations of vasodilating PGI_2_ when compared to control isolated vessels that were not exposed to EGCG. The increase in PGI_2_ concentrations resulting from EGCG was inhibited by pretreatment with the COX-1 selective inhibitor SC560 only, but not by COX-2 selective inhibition. The results we obtained with ECGC may therefore improve our understanding of the effects of cyclooxygenase inhibitors (both COXIBs and traditional NSAIDs) on endothelial cells as these cells appear to change their ability to synthesize the vasoprotective PGI_2_ as a result of COX-1 and/or COX-2 activity depending on the presence of oxidative stress and hydrogen peroxide. Moreover, we describe for the first time a possible link between hydrogen peroxide-mediated vasodilation and PGI_2_, a result consistent with the notion that COX enzymes are bi-functional proteins endowed with both cyclooxygenase (COX) and peroxidase (POX) activities, and that hydroperoxides are required for the first heme oxidation at the POX site of the enzyme [6].

Overall, these findings also provide new insights into the homeostatic mechanisms of the endothelium responses to ROS and H_2_O_2_ and support COX-1 as the main source of endothelial PGI_2_ under altered oxidative tone conditions, suggesting that it might be important to reappraise the impact of cyclooxygenase inhibitors on vascular PGI_2_ production in patients undergoing significant oxidative stress.
